# Global burden and health inequalities of drug use disorders in adolescents and young adults from 1992 to 2021

**DOI:** 10.3389/fpubh.2025.1659675

**Published:** 2025-12-05

**Authors:** Rongrong Bao, Huiqi Sun, Xinrui Zhang, Zhihui Zhao, Junhao Feng, Wenkai Jiang

**Affiliations:** 1 The First School of Clinical Medicine, Lanzhou University, Lanzhou, China; 2 The Second Clinical Medical School, Lanzhou University, Lanzhou, China; 3 Department of General Surgery, The First Affiliated Hospital of Xi'an Medical University, Xi'an, China

**Keywords:** Global Burden of Disease, drug use disorders, prevalence, disability-adjusted life year, health inequality

## Abstract

**Background:**

Drug use disorders (DUDs) are serious medical issues worldwide, especially in adolescents and young adults. This study aims to assess the global pattern and trends in disease burden due to DUDs among adolescents and young adults and its health inequalities from 1992 to 2021.

**Methods:**

We downloaded data on the prevalence and disability-adjusted life years (DALYs) due to DUDs among people aged 15–39 years from the Global Burden of Disease 2021. Age-period-cohort (APC) model was used to assess the time trend of disease burden. The slope index and the concentration index were calculated to assess cross-national health inequalities.

**Results:**

There were approximately 40.2 [95% uncertainty interval (UI): 34.7–47.7] million cases of DUDs among adolescents and young adults worldwide. The total burden of DUDs in adolescents and young adults measured in DALYs was 10.2 (95% UI: 8.2–12.3) million in 2021, an increase of 38.2% from 1992. In the APC model, the age effect showed that people aged 35–39 were at the lowest risk, and the highest risk existed in people aged 25–29 years; period effects showed that there was a decline first and then a rising risk of prevalence for both sexes; birth effects presented a rising first and then a declining risk of prevalence in successive birth cohorts. The slope index increased from 195.9 [95% confidence interval (CI): 166.1–225.7] in 1992 to 228.7 (95% CI: 190.4–267) in 2021. The concentration index also increased from 0.24 (95% CI: 0.19–0.29) in 1992 to 0.5 (95% CI: 0.36–0.64) in 2021.

**Conclusion:**

Disease burdens due to DUDs among adolescents and young adults suggest that more measures should be taken to limit drug abuse. We should pay attention to body health and substance use in adolescents and young adults.

## 1 Introduction

Drug use disorders (DUDs) remain a medical issue worldwide. The increase in DUD prevalence has led to a tremendous burden on society and a sharp rise in death (1, 2). DUDs display a consistent pattern of pathological drug or substance use, which leads to recurrent negative social outcomes attributed to drug consumption. These consequences include the inability to fulfill responsibilities at work, with family or school, conflicts in interpersonal relationships, and encountering legal issues (3). Moreover, drug users are more prone to suicide and depression, and also have an increased risk of infectious diseases (4–6). As a result of the succession of adverse personal and societal consequences triggered by DUDs, it has become an indisputable crisis in public health.

Adolescence is an important stage in the development of human psychology and behavior. People in this period have a sense of self-choice and self-control. However, adolescents and young adults are susceptible to the negative influence of behavioral factors. In 2019, DUDs were the 18th leading cause of disability-adjusted life-years (DALYs) at the global level among adolescents (7). During the past few decades, DUD cases have increased by 33.5% globally, and teenagers aged 15–19 years have the highest incidence rate (8). The vulnerability of adolescents and young adults to DUDs is often affected by a range of environmental and psychosocial risk factors. These include, but are not limited to, early life adversity, peer substance use, and the co-occurrence of mental health conditions. Most adults with DUDs initiated drug use during their teenage years (9). Adolescence and young adulthood are crucial stages marked by extensive transformations in physical, cognitive, emotional, social, and behavioral aspects (10). Information on drug use patterns among youths is limited, and studies that specifically focus on the health inequalities in the disease burden of DUDs across different regions are lacking. In this case, it is important to explore epidemiological data on DUDs among adolescents and young adults.

In this study, we used data from the Global Burden of Disease (GBD) 2021 to explore the burden and health inequalities of drug use disorders in adolescents and young adults from 1992 to 2021. We describe the prevalence and disability-adjusted life years (DALYs) by region, country, sex and age group and explore the change trends using age-period-cohort (APC) model. We also analyze patterns of cross-country inequalities. This study can help design more effective policies and methods to prevent drug use disorders tailored to different regions or countries.

## 2 Methods

### 2.1 Data sources

Data including annual counts and rates of prevalence and DALYs among people aged 15–39 years were downloaded from the GBD 2021 (http://ghdx.healthdata.org/gbd-results-tool). GBD 2021 included 371 diseases and injuries in 21 GBD regions, 204 countries/territories and five sociodemographic index (SDI) quintiles, from 1990 to 2021 (11). The estimation process is based on censuses, disease registries, health service use, disease notifications and other sources. In GBD 2021, DUDs included opioid use, cocaine use, amphetamine use, cannabis use and other drug use, which were from vital registration and surveillance sources (11). Drug use disorders were identified according to the International Classification of Diseases, version 10 as follows: F11–F19.99, P96.1, R78.1–R78.9, and Z81.2–Z81.4. Adolescents and young adults are defined as individuals aged 15–39 years (12).

### 2.2 SDI

The SDI, which stands for the sustainable development index, serves as a reliable measure for assessing the progress of a nation or locality. Its computation entails taking into account various factors including the fertility rate of women below the age of 25, the mean educational attainment of individuals aged 15 years and above, and the per capita income (13). More information about the calculation of SDI be found in the publication provided by the GBD 2021 Diseases and Injuries Collaborators (11). According to the critical value, all countries/territories can be divided into 5 levels: high, high-middle, middle, low-middle and low. The SDI values of all regions and countries/territories from 1990 to 2021 can be downloaded at: https://ghdx.healthdata.org/record/global-burden-disease-study-2021-gbd-2021-socio-demographic-index-sdi-1950–2021.

### 2.3 APC analysis

We used APC models to estimate the effects of age, period and birth cohort on the prevalence of DUDs in adolescents and young adults from 1992 to 2021.

The APC model has been widely used in GBD studies (14, 15). In the APC model, net drift and local drifts represent the overall log-linear trend of the prevalence rate of DUDs, and the log-linear trend for each age group, respectively (16). The age effect refers to the age-specific prevalence rate of DUDs at different ages (longitudinal age curve); the period effect refers to the effect of temporal changes on outcomes across all age groups, which captures the impact of external factors that simultaneously affect all age groups during a specific time interval (period relative risk); and the cohort effect refers to the changes in outcomes among participants with the same birth cohorts, which can signify the risk inherent to specific birth cohorts (cohort relative risk). In this study, we choose six 5-year periods (1992–1996, 1997–2001, ……, 2017–2021, reference: 2002–2006). As the relationships among age, period, and cohort are perfectly linear, there were 10 birth cohorts in this model (1957, 1962, ……, 2002, reference: 1977). We calculated the estimated parameters from the Age Period Cohort Web Tool (https://analysistools.cancer.gov/apc/) from the National Cancer Institute (17).

### 2.4 Cross-national health inequalities

We also quantify the extent to which the global burden of DUDs is inequitably distributed across nations, reflecting broader socioeconomic disparities. Health inequalities are crucial for understanding whether the DUD burden disproportionately affects more affluent or more disadvantaged societies, and how this pattern may be related to differing national capacities for prevention, treatment, and enforcement. We calculate the slope index and the concentration index to assess absolute and relative health inequalities, respectively. The slope index is an absolute measure of inequality that takes into account all population subgroups. It can represent the difference in DUD burden between the most-advantaged (the country with the highest SDI) and the most-disadvantaged (the country with the lowest SDI) countries, based on the regression model (18). The concentration index is a relative measure of inequality that is calculated via numerical integration under the Lorenz curve, which is defined as the cumulative proportion of DUD DALYs and the cumulative population distribution ranked by the SDI (19). The greater the absolute values of the slope index and the concentration index are, the higher the level of inequality (20).

### 2.5 Data analysis

The estimates for all the metrics are computed with the mean estimate across 500 draws, and 95% uncertainty intervals (UIs) are given as the 2.5th and 97.5th percentiles of that distribution (21). All rates are reported per 100,000 people. Data visualization was carried out in R software (version 4.4.1).

## 3 Results

### 3.1 Overview of the global burden of DUDs

**Figure 1:**
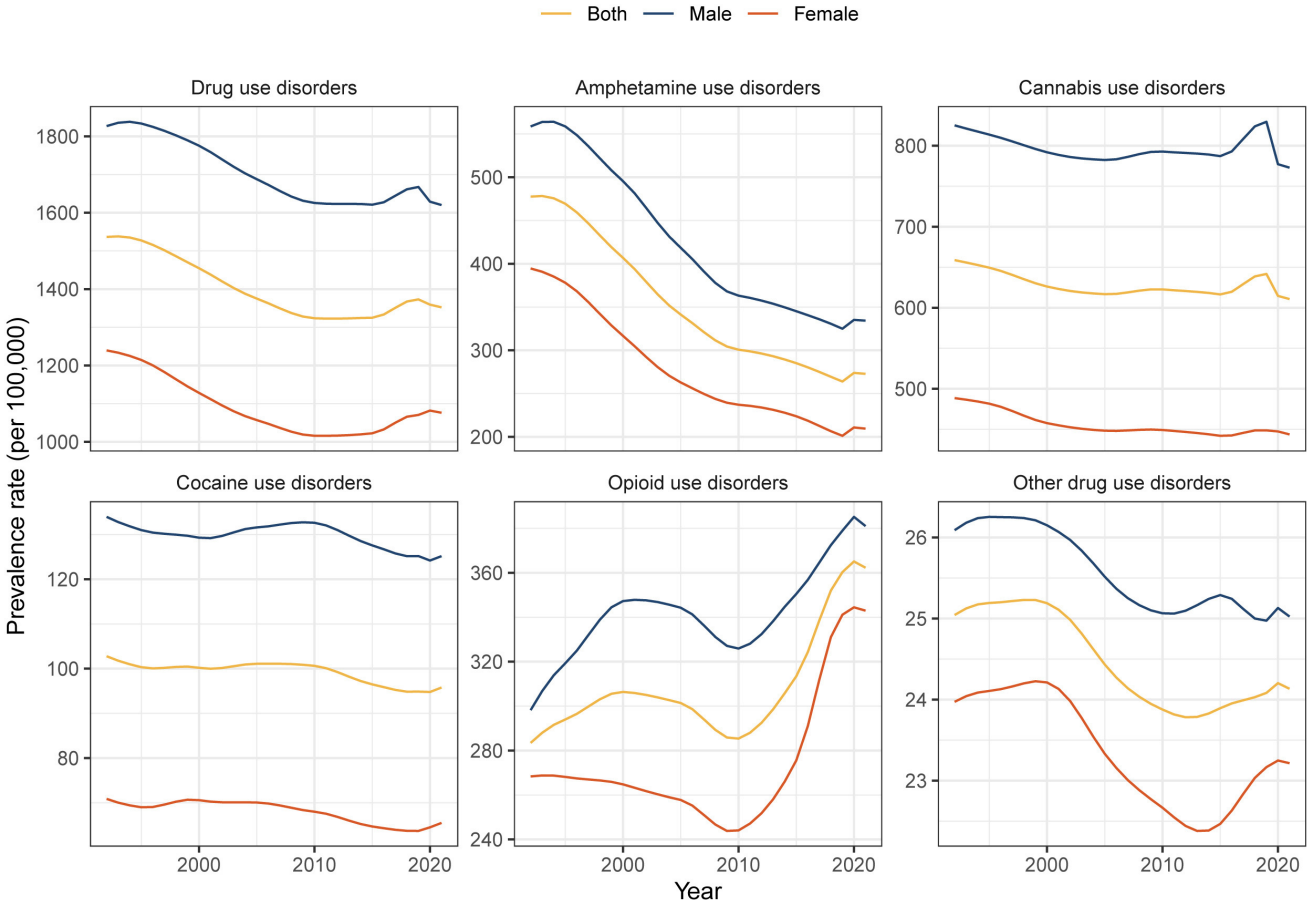
Global prevalence of drug use disorders by sex from 1992 to 2021.

According to the GBD 2021, there were approximately 40.2 (95% UI: 34.7–47.7) million cases of DUDs among adolescents and young adults in 2021 worldwide, of which 60.8% were males. The total burden of DUDs in adolescents and young adults measured in DALYs was 10.2 (95% UI: 8.2–12.3) million in 2021, an increase of 38.2% from 1992 (7.4, 95% UI: 5.8–8.9; million). The prevalence rate was 1,352.2 (95% UI: 1,167.3–1604.9) in 2021, with a net drift of −0.47% [95% confidence interval (CI): −0.58 to −0.36] from 1992 to 2021. The DALY rate was 342.9 (95% UI: 275.1–412.1) in 2021, with a percentage change of 4.6% during the past 30 years (Figure 1, Table 1). The temporal trends in the prevalence of DUDs among adolescents and young adults in the five SDI quintiles are shown in Supplementary Figure S1. Males suffered a heavier burden of DUDs than females, and the age-stratified burden of DUDs measured in DALYs was greatest in the 25–29 years age group in 2021 (Supplementary Figure S2). Among the different age subgroups, the greatest decline in both males (local drift: −0.6%, −0.79% to −0.41%) and females (−0.81%, −1.07% to −0.55%) was observed in 15–19 years group.

**Table 1:** Global total counts and rates of prevalence and DALYs of drug use disorders in 2021.

**Cause**	**Number of cases (thousand)**	**Prevalence rate (per 100,000)**	**Number of DALYs (thousand)**	**DALY rate (per 100,000)**
Drug use disorders	40,226.4 (34,724.7–47,744.8)	1,352.2 (1,167.3–1,605)	10,201.3 (8,184.1–12,258.6)	342.9 (275.1–412.1)
Amphetamine use disorders	8,115.7 (5,704.3–11,030.4)	272.8 (191.8–370.8)	1,345.6 (876.6–1,946.2)	45.2 (29.5–65.4)
Cannabis use disorders	18,168.3 (13,486.6–25,017.7)	610.7 (453.4–841)	525.6 (310.1–832.3)	17.7 (10.4–28)
Cocaine use disorders	2,849.2 (2,130.2–3,777.6)	95.8 (71.6–127)	705.9 (544.2–925.2)	23.7 (18.3–31.1)
Opioid use disorders	10,777.8 (9,125.5–12,785.6)	362.3 (306.8–429.8)	7,111.5 (5,675.6–8,638.5)	239.1 (190.8–290.4)
Other drug use disorders	717.9 (548.5–939.2)	24.1 (18.4–31.6)	512.7 (467.5–564.6)	17.2 (15.7–19)

DALY, disability-adjusted life year.

**Figure 2:**
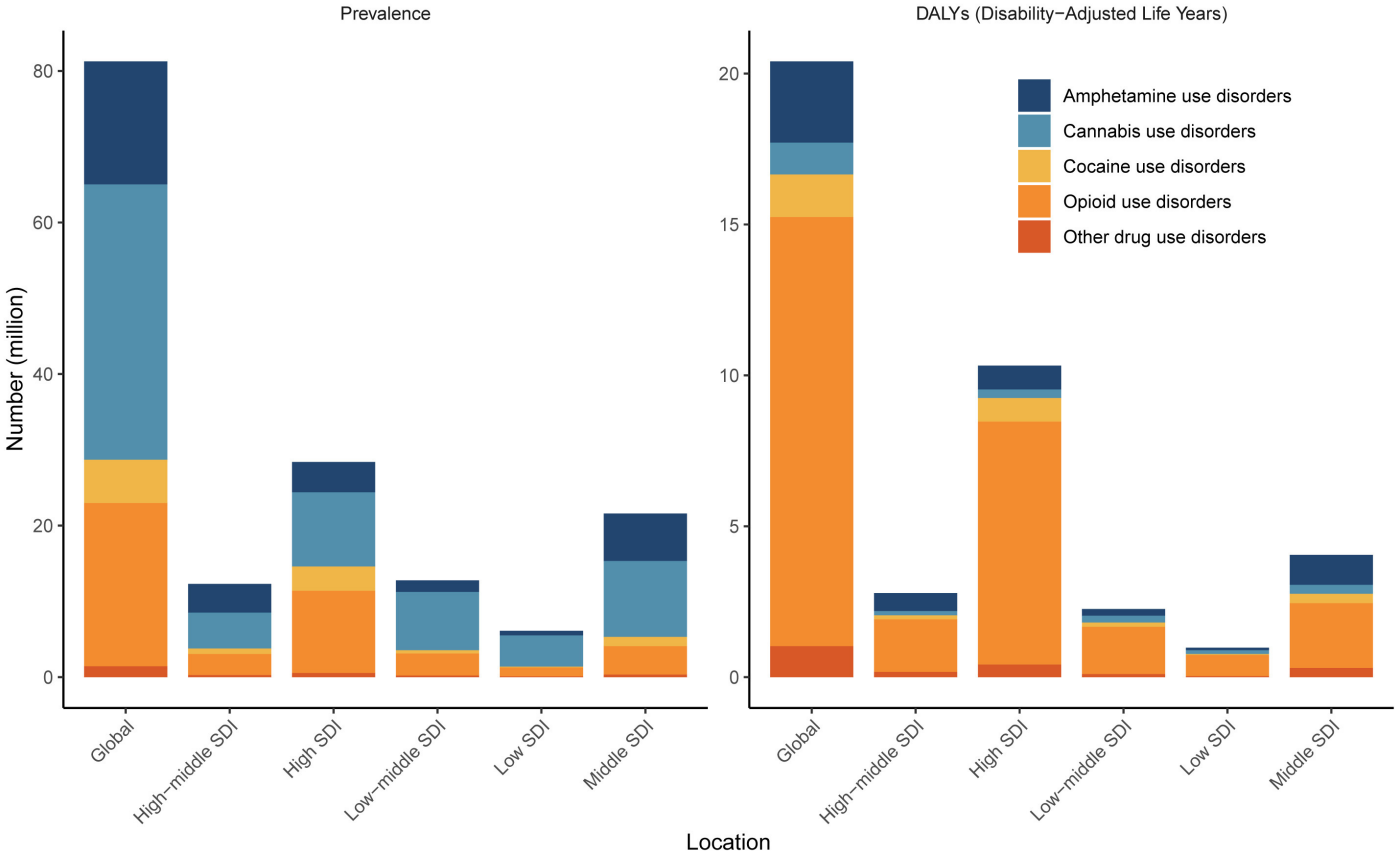
Number of cases and DALYs of five types of drug use disorders in 2021 according to SDI. DALY, disability-adjusted life-year. SDI, sociodemographic index.

Among the five type subgroups, cannabis use disorder was the most common type of DUDs worldwide, followed by opioid use disorders. However, opioid use disorders caused the greatest burden, as measured by DALYs (Figure 2).

### 3.2 Spatial distribution of the burden of DUDs

**Figure 3:**
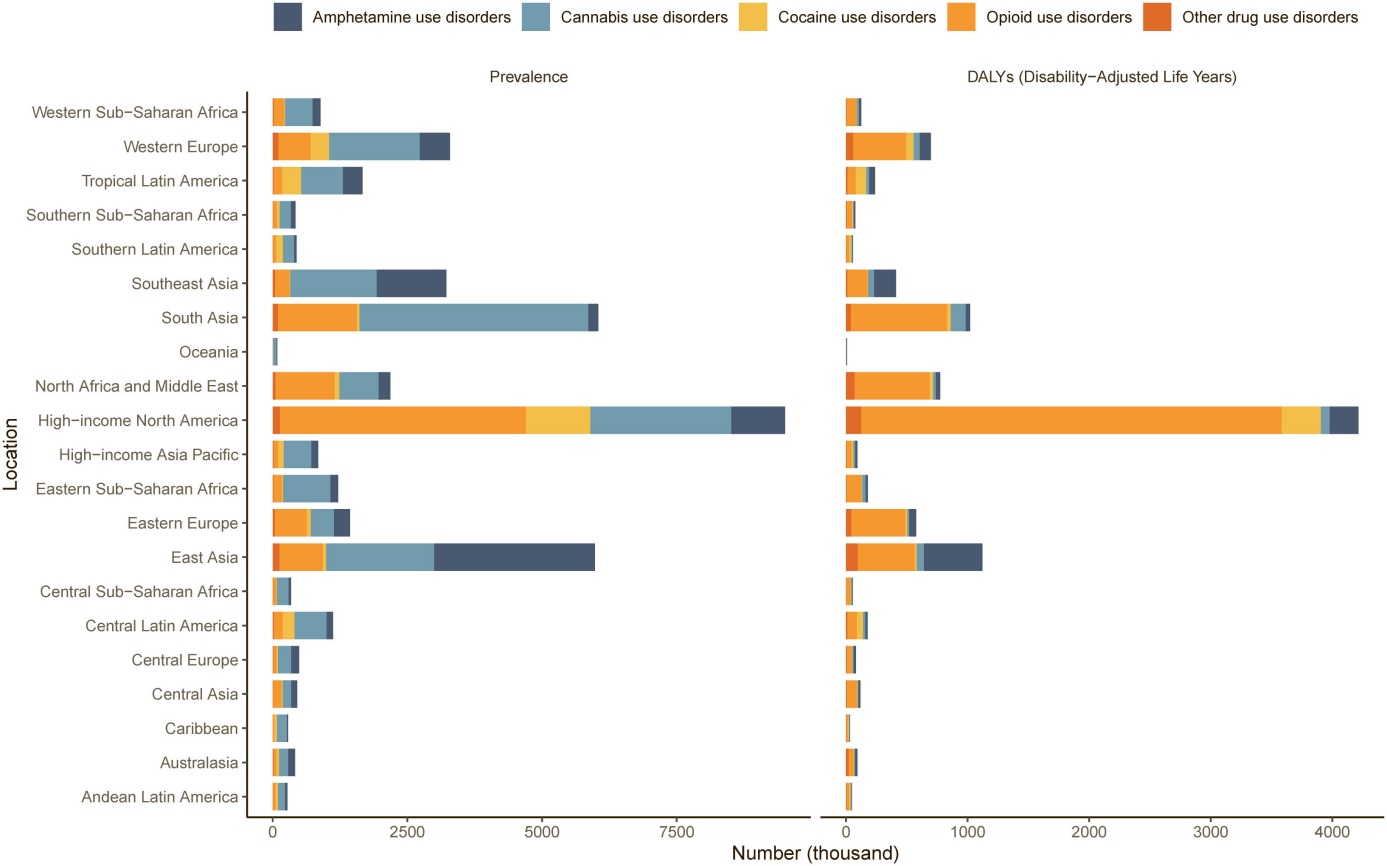
Number of cases and DALYs of five types of drug use disorders in 2021 across 21 regions. DALY, disability- adjusted life-year.

**Figure 4:**
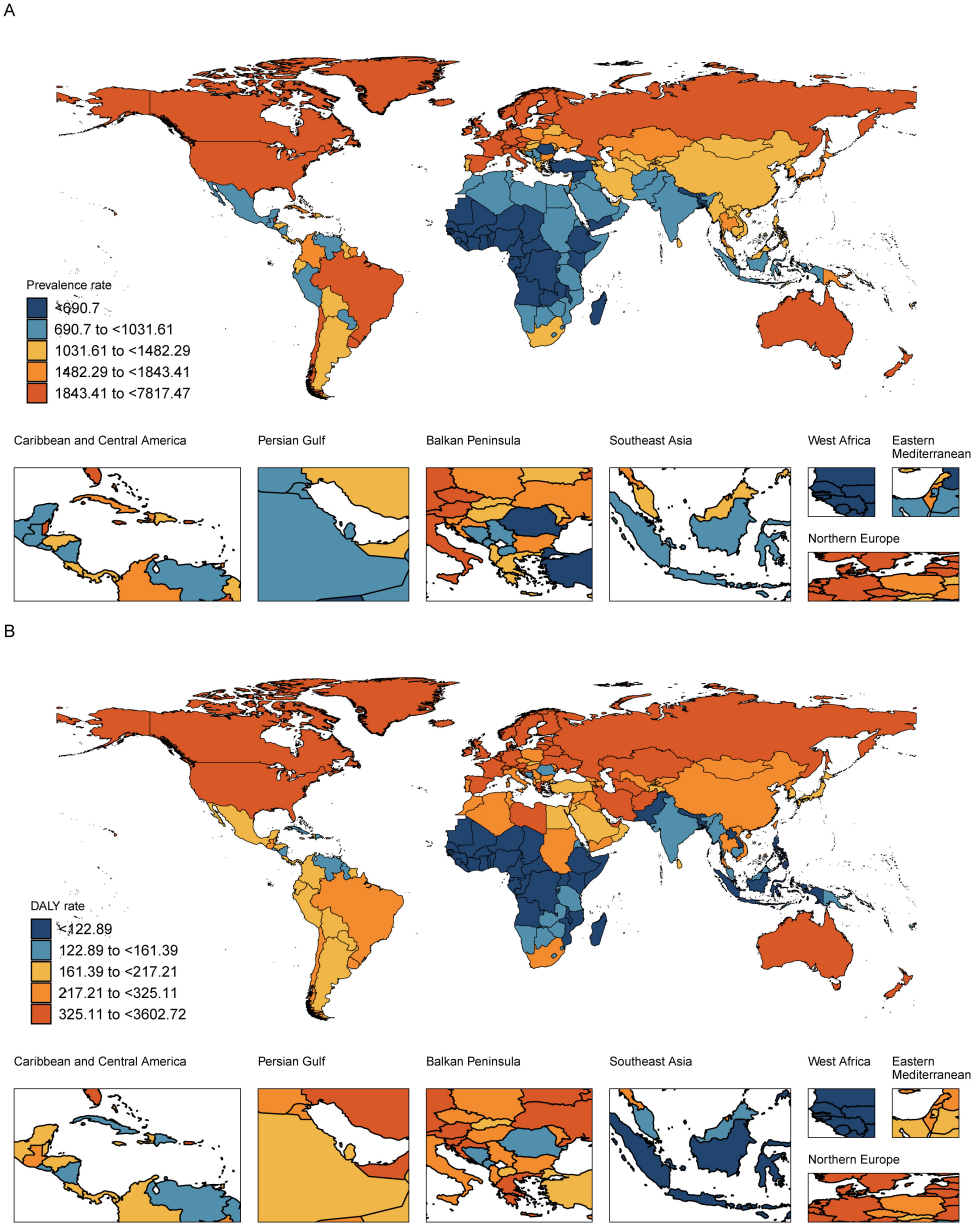
Spatial distribution of the **(A)** prevalence and **(B)** DALY rates of drug use disorders by country or territory in 2021. DALY: disability- adjusted life-year.

At the regional level, high-income North America had the highest number of cases and DALYs of DUDs among young adults and adolescents (Figure 3). High-income North America, Australasia and Western Europe were the three regions with the highest prevalence rates of DUDs in young adults and adolescents, with high-income North America having the highest net drift (2.12%, 95% CI: 1.97%−2.27%; Supplementary Figure S3). The highest DALY rates were also observed in high-income North America and Australasia (Table 2). At the national level, the countries with the highest prevalence rates were United States of America, Canada and New Zealand, and the countries with the highest DALY rates were United States of America, Canada and Estonia (Figure 4). The 10 countries with the highest and lowest prevalence rates of DUDs in adolescents and young adults globally in 2021 are shown in Supplementary Tables S1, S2.

**Table 2:** Total counts and rates of prevalence and DALYs of drug use disorders across 21 regions in 2021.

**Location**	**Number of cases (thousand)**	**Prevalence rate (per 100,000)**	**Number of DALYs (thousand)**	**DALY rate (per 100,000)**
Andean Latin America	274.8 (227.2–338.7)	1,014.9 (838.9–1,250.7)	48.8 (37.3–62.2)	180.3 (137.6–229.8)
Australasia	407.8 (359.9–466.9)	3,894.5 (3,437–4,459.1)	92.9 (76–110.7)	886.7 (725.5–1,057.5)
Caribbean	285 (211.4–383.5)	1,565.8 (1,161.2–2,106.9)	33 (24.5–43.2)	181.2 (134.5–237.3)
Central Asia	451.9 (374–548.1)	1,208.7 (1,000.3–1,466.1)	117.7 (88.8–145.6)	314.7 (237.6–389.5)
Central Europe	487.5 (414.9–581.2)	1,392 (1,184.8–1,659.7)	81.7 (64–98.6)	233.3 (182.9–281.6)
Central Latin America	1,114.4 (955.2–1,320)	1,101.5 (944.2–1,304.8)	177.3 (135.2–222.9)	175.3 (133.6–220.3)
Central Sub-Saharan Africa	341.2 (256.4–470.1)	630.8 (474–869)	55 (39.7–70.9)	101.8 (73.3–131.1)
East Asia	5,955.3 (4,908.4–7,273.9)	1,243.2 (1,024.6–1,518.4)	1,121.4 (838.5–1,410.8)	234.1 (175–294.5)
Eastern Europe	1,424.2 (1,240.2–1,656.1)	2,152.2 (1,874.2–2,502.7)	577.2 (484.1–677.6)	872.3 (731.6–1,024)
Eastern Sub-Saharan Africa	1,212.8 (894.2–1,681.3)	692.3 (510.4–959.7)	179.5 (137.8–227.1)	102.5 (78.7–129.6)
High-income Asia Pacific	841.9 (679.1–1,082.4)	1,665.7 (1,343.6–2,141.7)	93.3 (66.5–123.2)	184.5 (131.6–243.8)
High-income North America	9,256.1 (8,272.9–10,375.5)	7,514.1 (6,715.8–8,422.8)	4,214.6 (3,506.2–4,935)	3,421.4 (2,846.3–4,006.2)
North Africa and Middle East	2,176.6 (1,848.9–2,578.5)	856 (727.2–1,014.1)	775.2 (610.6–952)	304.9 (240.1–374.4)
Oceania	85.9 (60.9–118.6)	1,524.1 (1,080.1–2,104.3)	8 (5.6–10.8)	141.9 (99.1–191.1)
South Asia	6,032.7 (4,733–7,890.2)	762.7 (598.4–997.6)	1,019.5 (773.7–1,316.7)	128.9 (97.8–166.5)
Southeast Asia	3,213.1 (2,545.4–4,098.1)	1,158.6 (917.8–1,477.7)	410.6 (293.9–546.7)	148 (106–197.1)
Southern Latin America	441.8 (393.3–505)	1,712.6 (1,524.6–1,957.8)	57 (40.2–74.9)	221 (156–290.3)
Southern Sub-Saharan Africa	421.2 (339.3–524.8)	1,237.6 (997–1,542)	76.9 (60.8–93.9)	225.9 (178.7–275.8)
Tropical Latin America	1,657.6 (1,372.6–2,001.5)	1,877 (1,554.3–2,266.5)	238.3 (177.6–298.9)	269.8 (201.1–338.4)
Western Europe	3,258.9 (2,892.6–3,726.4)	2,511.3 (2,229–2,871.5)	698.2 (571.4–821.7)	538 (440.3–633.2)
Western Sub-Saharan Africa	885.7 (705.5–1,142.8)	463.2 (369–597.7)	125.4 (86.7–168)	65.6 (45.4–87.9)

DALY, disability-adjusted life year.

### 3.3 APC model

**Figure 5:**
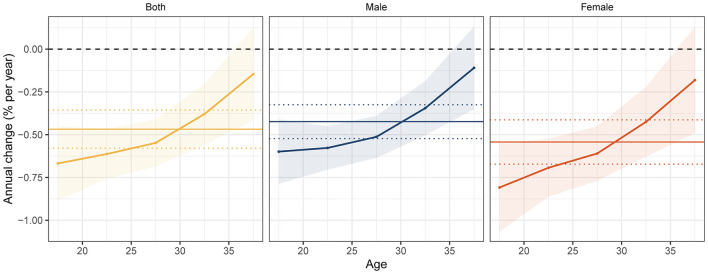
Local drift and net drift values for drug use disorder prevalence from 1992 to 2021.

**Figure 6:**
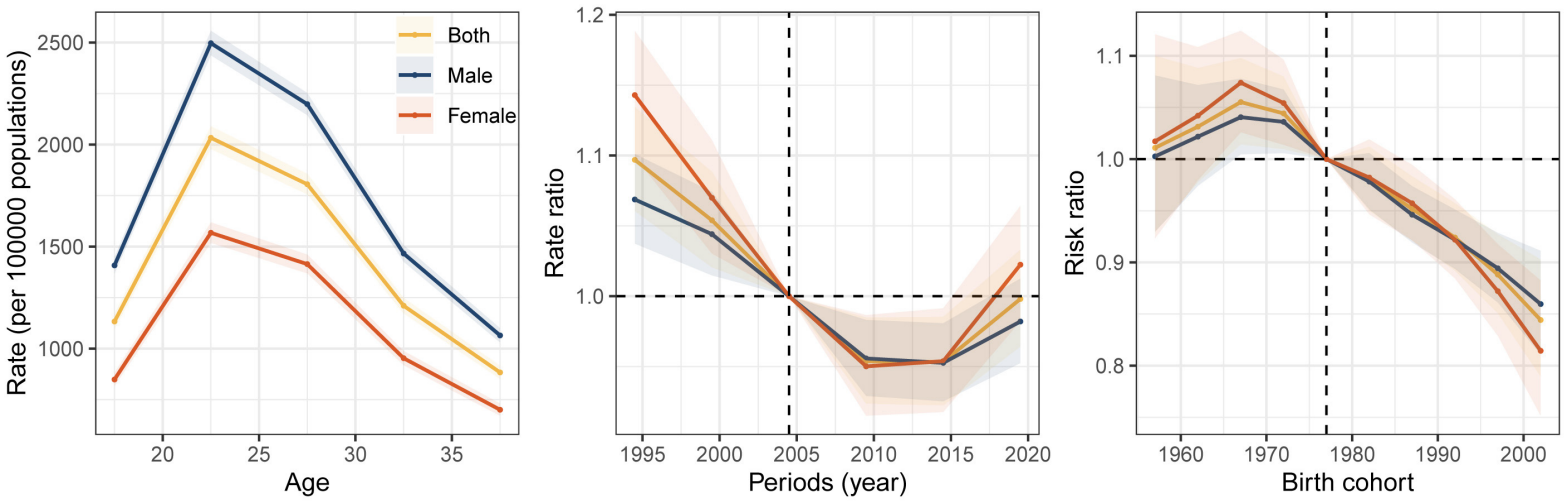
Age, period and cohort effects on drug use disorder prevalence.

The Local drift and net drift values for prevalence of DUDs in the APC model were shown in Figure 5. Figure 6 shows the APC model of DUD prevalence by sex. The longitudinal age curves revealed similar patterns in age effects for both sexes that the lowest risk existed in people aged 35–39 years and the highest risk existed in people aged 25–29 years. Period effects presented a decreasing risk and then an increasing risk of prevalence for both sexes. Compared with individuals in the reference period, the relative period risk for individuals in the 2017–2021 period was 0.98 (95% CI: 0.95–1.01) for males and 1.02 (95% CI: 0.98–1.06) for females. In the birth cohort, the risk of prevalence first increased but then decreased. Compared with individuals born in the reference cohort, the relative cohort risk for individuals born in the 1997–2006 cohort was 0.86 (95% CI: 0.81–0.91) for males and 0.81 (95% CI: 0.75–0.88) for females. The age, period, and cohort effects in the five SDI quintiles are shown in Supplementary Figures S4–S6. The rate ratios (2017–2021) were higher than those in the reference period in high-SDI, low-middle-SDI and low-SDI-regions in period effect, and rate ratio (1997–2006) was higher than that in the reference period in high-SDI region in cohort effect.

### 3.4 Cross-national health inequality

The slope indexes were 195.9 (95% CI: 166.1–225.7) and 228.7 (95% CI: 190.4–267) DALYs per 100,000 population in 1992 and 2021, respectively. This finding indicates that the inequality in the burden of DUDs in adolescents and young adults between high-SDI countries and low-SDI countries increased during this time. The change in the slope index varied across the different DUD types. Between 1992 and 2021, the slope index for cannabis use disorders decreased, whereas amphetamine use disorders, opioid use disorders and other drug use disorders increased (Table S3).

**Figure 7:**
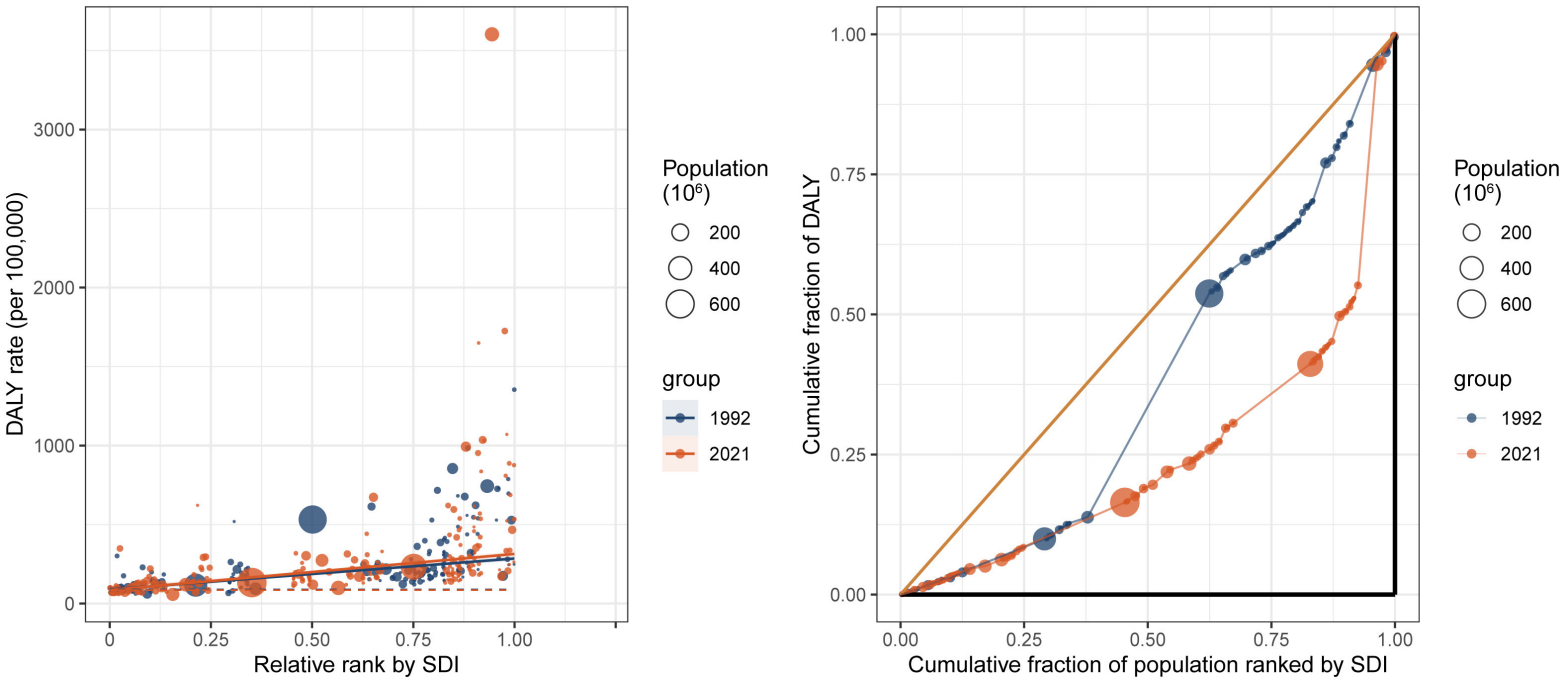
SDI related health inequality in the DALY of DUDs. SDI, sociodemographic index.

The concentration index also increased between 1992 (0.24, 95% CI: 0.19–0.29) and 2021 (0.5, 95% CI: 0.36–0.64; Figure 7). Regarding the various DUDs, the SDI-related concentration index increased for amphetamine use disorders, opioid use disorders and other drug use disorders, and decreased for cannabis use disorders and cocaine use disorders (Supplementary Table S4).

## 4 Discussion

DUDs are serious medical problems worldwide. However, detailed information on the burden of DUDs among adolescents and young adults is still lacking. Our study reported the temporal trends of DUD burden among people aged 15–39 years at the global, regional, and national levels and assessed its health inequalities, which supplements previous publications. The prevalence and DALY rate of DUDs have notably decreased since 1992, and these trends vary considerably by sex, region and country. High-SDI regions had greater burdens of DUDs, and cross-national health inequality, as measured by the slope index and concentration index, significantly increased between 1992 and 2021. These results suggest that we should pay attention to the DUD burden among adolescents and young adults. Because adolescents and young adults are at a critical stage in both physical and mental development, targeted science education and prevention measures for adolescents and young adults are needed.

Cannabis ranks as the third most commonly used substance worldwide, after alcohol and tobacco (22). Approximately 3.9% of adults worldwide have experimented with cannabis (22). The initiation of cannabis consumption predominantly takes place during late adolescence, and there is an upward trend in cannabis usage among young individuals (23). Adolescent cannabis users may be at high risk for psychotic symptoms and neurocognitive impairments (23). Cannabis use in people younger than 18 years is associated with an increased risk of car accidents, antisocial behavior and polysubstance use (22). In 2019, 1.6 million Americans older than 12 years were estimated to have opioid use disorders and opioid overdose contributed to nearly 69 thousand deaths in the USA in 2020 (24). Additionally, there was a two-fold increase in the number of deaths caused by an overdose of cocaine, and there were one million individuals with cocaine use disorder in the United States in 2017 (25). Given the current high prevalence of DUDs and the increasing number of deaths each year, public health measures are necessary to reduce the disease burden of DUDs.

Similar to previous studies, our study demonstrated that high-income North America experienced a heightened DUD burden and that the cross-national health inequalities have increased over the past three decades. The DUD-related burden has increased in the US (26). People in high-SDI regions or countries may be more inclined to use drugs (27). However, people in low-income regions may face additional socio-economic disadvantages, such as poverty, a lack of education and poor medical care, making them highly vulnerable to mental health problems and DUDs (26). It is imperative that we allocate essential resources and undivided attention toward tackling all facets of the global drug predicament, including the provision of evidence-based care.

The rapid progression of physiological growth can impact cognitive reasoning and emotional regulation during puberty. In addition, adolescents are achieving significant developmental and life milestones, such as completing their education, embarking on a career, and striving for independence. Drug use in adolescence is linked to a somewhat elevated likelihood of experiencing depression in young adulthood (28). Adolescence and early adulthood are crucial stages when drug usage habits may form. These periods are pivotal for preventing the initiation of such behaviors, curbing the progression toward excessive drug consumption, and intervening in addressing existing substance abuse issues. Excessive cannabis consumption during adolescence has detrimental effects on cognitive functions such as memory, learning, recall, attention, problem-solving, reasoning proficiency, and intelligence. In addition to personal factors, the prognosis is worsened by accessibility to substances, socioeconomic disadvantage, peer influence, and problems in the family (29). Therefore, well-designed and implemented prevention programs for adolescents may significantly reduce the disease burden of DUDs. The allure of substance use among youth should be diminished, and access to substances to render them harder to acquire or partake in should be restricted.

While high-SDI countries generally possess more robust healthcare systems and resources, they face disproportionately high prevalence rates of DUDs. It is essential to first enhance adolescents' mental health and emotional management skills through school- and community-based initiatives. For instance, the Life Skills Training implemented in Spain may prove effective for avoiding escalation of the consumption levels of problematic drugs (30). Moreover, exercise and athletic team participation worked synergistically in lowering drug use in students (31). Second, comprehensive support, including psychological therapy, vocational training, and educational assistance, should be made readily accessible. In contrast, countries with low SDI often face challenges such as resource scarcity and underdeveloped health systems. Therefore, efforts should focus on strengthening medical infrastructure and expanding service coverage. Moreover, higher prevalence rates of DUDs can be found in unemployed people (32). Thus, it is important to create more employment opportunities for young people.

Our study has several limitations. First, the low coverage of disease reporting institutions in underdeveloped regions led to inadequate data quality, particularly in countries where there was an absence of original, high-quality epidemiological research on drug use disorders. For regions or countries without data sources, GBD estimates heavily rely on the modeling process, predictive covariates and trends from neighboring countries, resulting in some uncertainty (33). Second, DUDs in GBD 2021 include other drugs, such as hallucinogens, sedatives and tranquilizers, but epidemiological data on these specific drugs are lacking. In the future, more attention should be given to the burden of DUDs in various countries and regions. It is imperative to obtain updated regional and national data to obtain precise information of DUDs in adolescents and young adults. The economic burden caused by DUDs should also be studied in depth.

## 5 Conclusion

In summary, despite a decline in the prevalence and DALYs of DUDs among adolescents and young adults between 1992 and 2021, there are still cross-national health inequalities in which countries with high SDI have a higher burden of DUDs. Further investigation is necessary to determine the causes of these changes to establish effective strategies and interventions to reduce the burden of DUDs in adolescents and young adults.

## Data Availability

Publicly available datasets were analyzed in this study. This data can be found at: http://ghdx.healthdata.org/gbd-results-tool.
