# Supplementary material for: Global burden and health inequalities of drug use disorders in adolescents and young adults from 1992 to 2021
Source: Front Public Health. 2025 Dec 5;13:1659675. doi: 10.3389/fpubh.2025.1659675 (PMC12715014; doi:10.3389/fpubh.2025.1659675)
Supplement: Supplementary file 1 [file Table_1.docx]

**Table S1** The 10 countries with the highest prevalence rate of DUDs in adolescents and young adults globally in 2021

| Location | Prevalence rate (95% UI) |
| --- | --- |
| United States of America | 7817.5 (6954 to 8778.6) |
| Canada | 4674.2 (4343.8 to 5063.2) |
| New Zealand | 4270.2 (3459.4 to 5413.6) |
| Estonia | 4142 (3840.5 to 4439.7) |
| United Kingdom | 4015.8 (3372.1 to 4854.2) |
| Australia | 3816.4 (3394.8 to 4361) |
| Greenland | 3444.2 (2889.2 to 4149.9) |
| Spain | 3085.2 (2847.9 to 3378.1) |
| Switzerland | 2731.8 (2311.9 to 3274) |
| Ireland | 2628.7 (2389.2 to 2920.7) |

UI: uncertainty interval.

**Table S2** The 10 countries with the lowest prevalence rate of DUDs in adolescents and young adults globally in 2021

| Location | Prevalence rate (95% UI) |
| --- | --- |
| Gambia | 484.6 (371.3 to 649) |
| Côte d'Ivoire | 478.9 (371 to 637.4) |
| Niger | 478.6 (360.1 to 648.3) |
| Chad | 470.1 (357.6 to 636.4) |
| Mali | 469.6 (352.6 to 638.7) |
| Guinea-Bissau | 467 (360.3 to 622.8) |
| Guinea | 457.6 (352.1 to 616.7) |
| Burkina Faso | 438.1 (349.9 to 578.8) |
| Nigeria | 431.7 (345 to 557.5) |
| Togo | 406.1 (334.7 to 509.2) |

UI: uncertainty interval.

**Table S3** Slope index for five type drug use disorders in 1992 and 2021

| Cause | Slope index in 1992 | Slope index in 2021 |
| --- | --- | --- |
| Amphetamine use disorders | 35.9 (28.2 to 43.6) | 42.3 (32.9 to 51.8) |
| Cannabis use disorders | 17.3 (13.0 to 21.6) | 13.8 (9.69 to 17.9) |
| Cocaine use disorders | 28.0 (21.2 to 34.7) | 28.8 (21.7 to 36.0) |
| Opioid use disorders | 112.6 (86.3 to 138.8) | 129.6 (98.8 to 160.4) |
| Other drug use disorders | 12.4 (9.7 to 15.1) | 17.4 (13.3 to 21.5) |

**Table S4** Concentration index for five type drug use disorders in 1992 and 2021

| Cause | Slope index in 1992 | Slope index in 2021 |
| --- | --- | --- |
| Amphetamine use disorders | 0.14 (0.06 to 0.22) | 0.44 (0.38 to 0.51) |
| Cannabis use disorders | 0.24 (0.19 to 0.29) | 0.18 (0.13 to 0.23) |
| Cocaine use disorders | 0.55 (0.45 to 0.65) | 0.55 (0.38 to 0.71) |
| Opioid use disorders | 0.25 (0.20 to 0.30) | 0.53 (0.36 to 0.70) |
| Other drug use disorders | 0.16 (0.08 to 0.25) | 0.50 (0.39 to 0.60) |
