# Supplementary material for: Global burden and health inequalities of drug use disorders in adolescents and young adults from 1992 to 2021
Source: Front Public Health. 2025 Dec 5;13:1659675. doi: 10.3389/fpubh.2025.1659675 (PMC12715014; doi:10.3389/fpubh.2025.1659675)
Supplement: Supplementary file 2 [file Data_Sheet_1.docx]

**Supplementary figures**

**
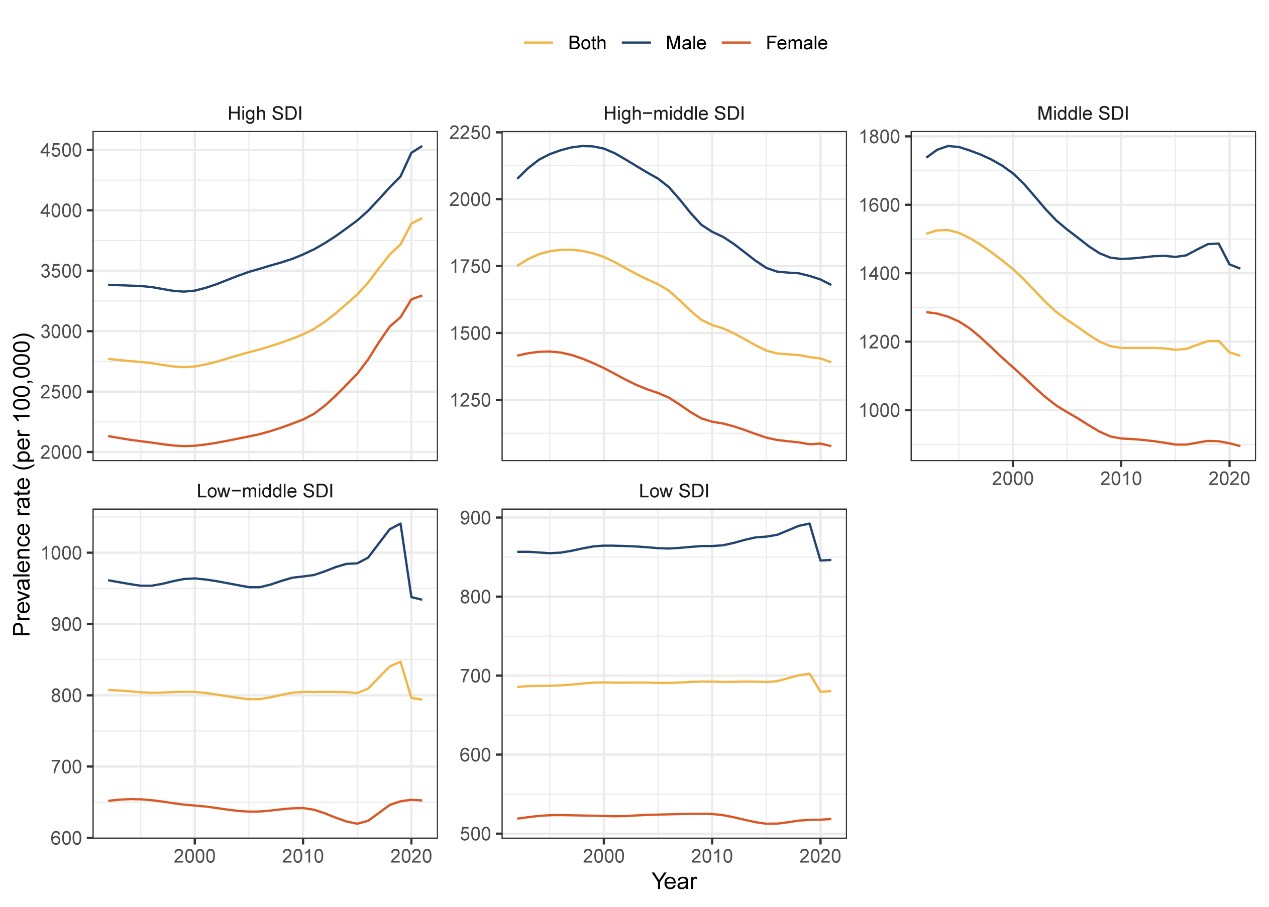
**

**Figure S1** Global prevalence of drug use disorders by sex in five sociodemographic index quintiles from 1992 to 2021


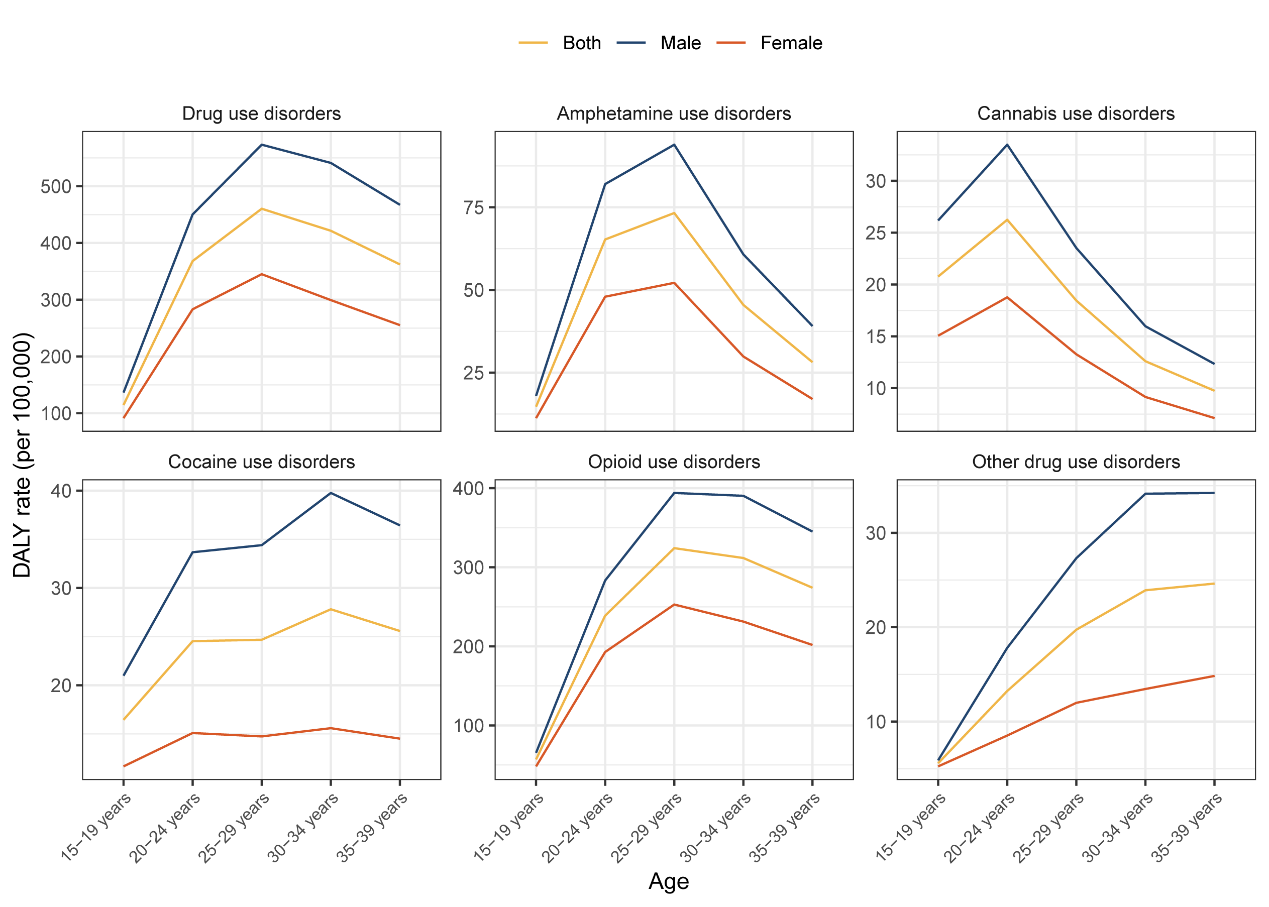


**Figure S2** Global prevalence of drug use disorders by sex in different age subgroups in 2021


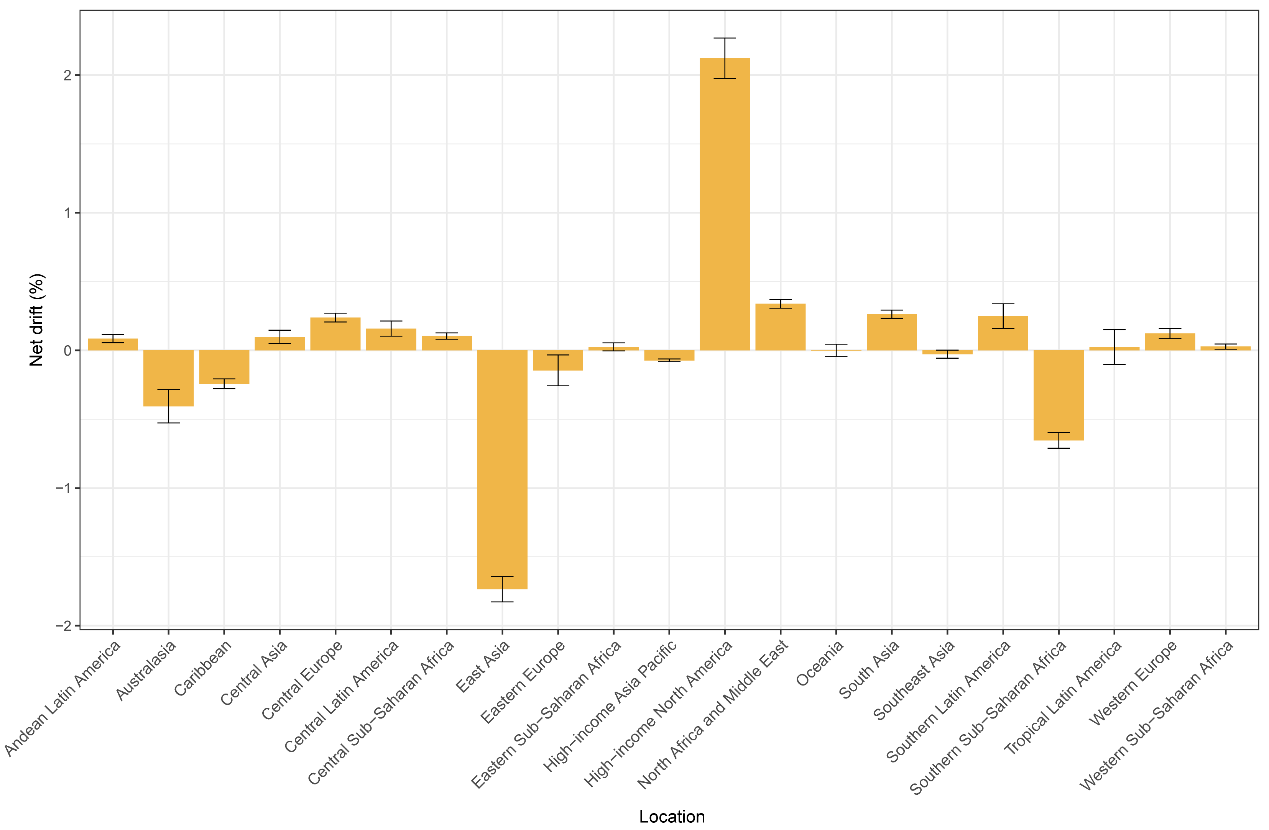


**Figure S3** Net drifts in age-period-cohort models of drug use disorder prevalence in 21 regions from 1990 to 2021

**
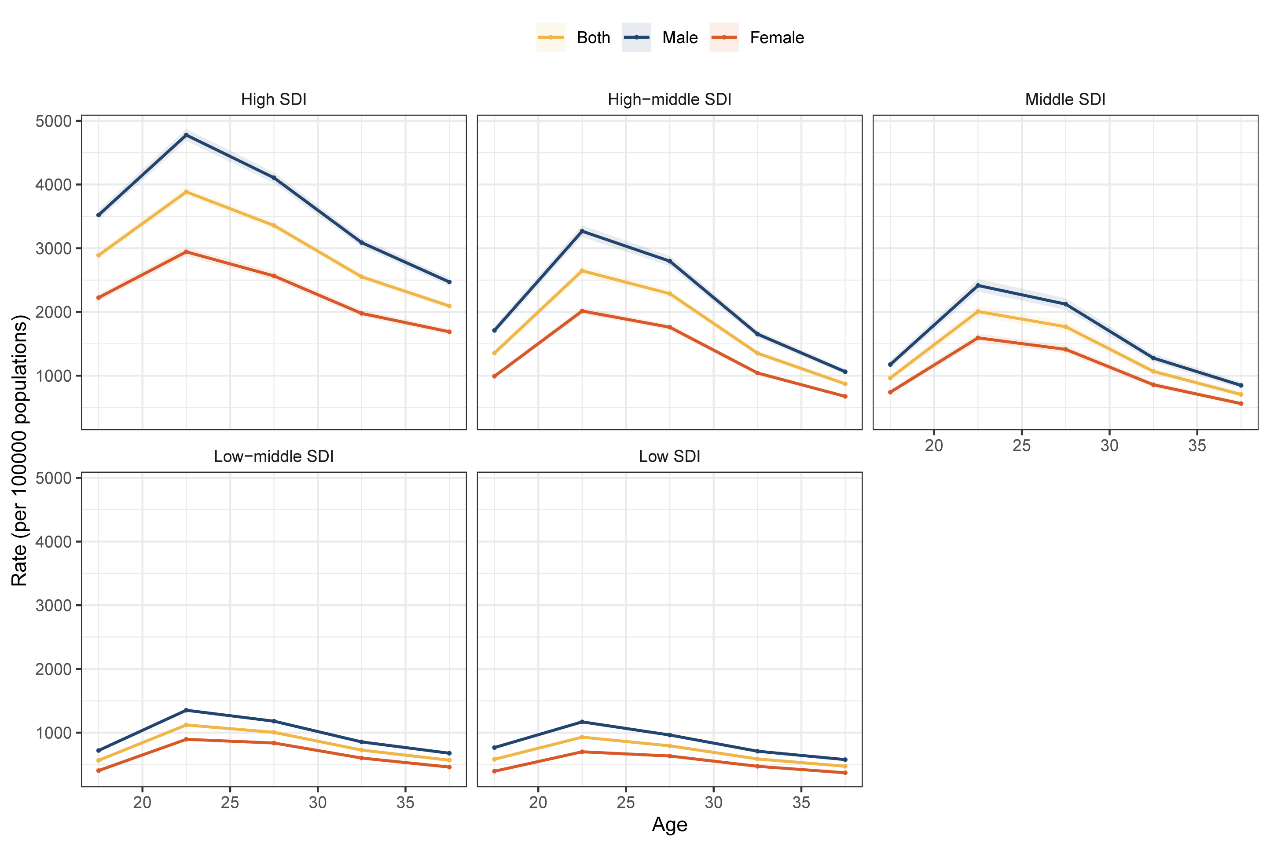
**

**Figure S4** Age effects in age-period-cohort models of drug use disorder prevalence in five sociodemographic index quintiles by sex

**
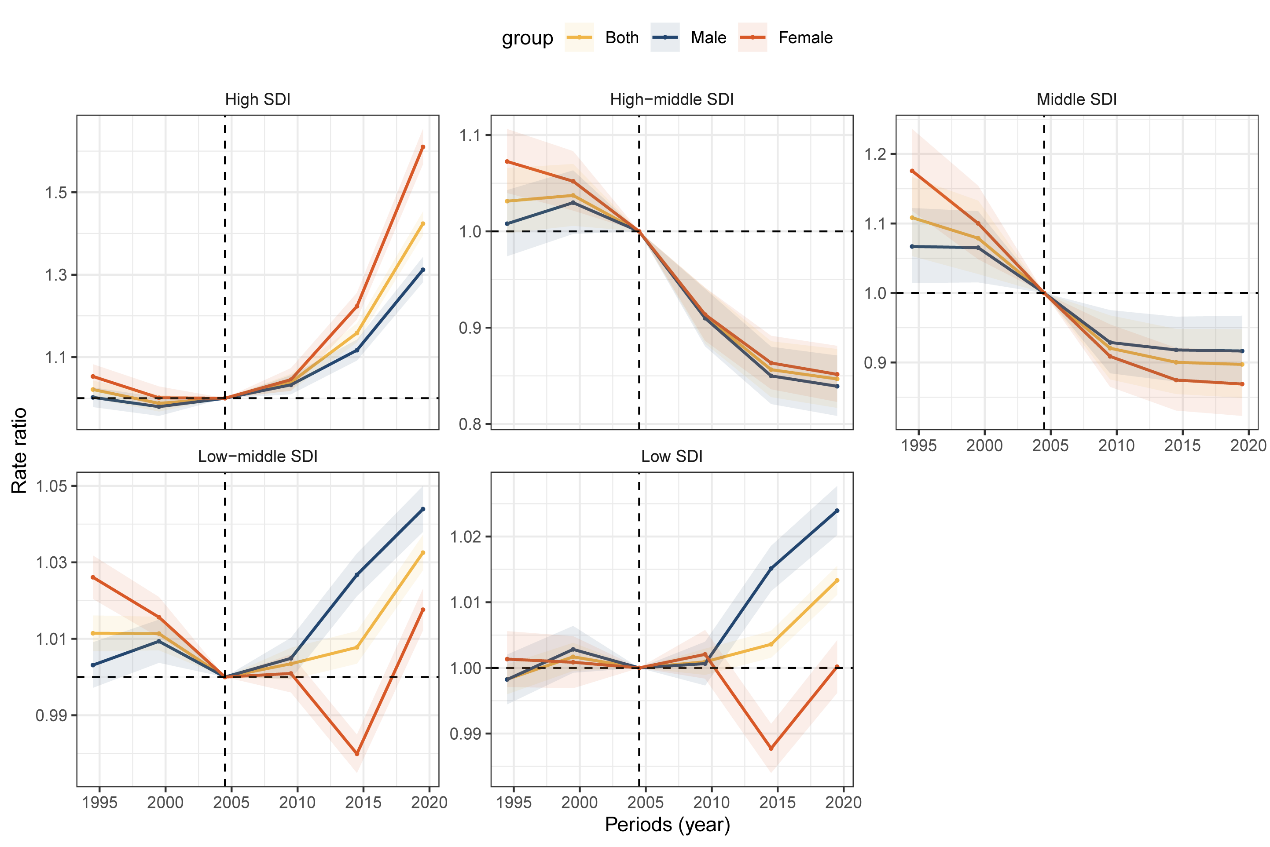
**

**Figure S5** Period effects in age-period-cohort models of drug use disorder prevalence in five sociodemographic index quintiles by sex

**
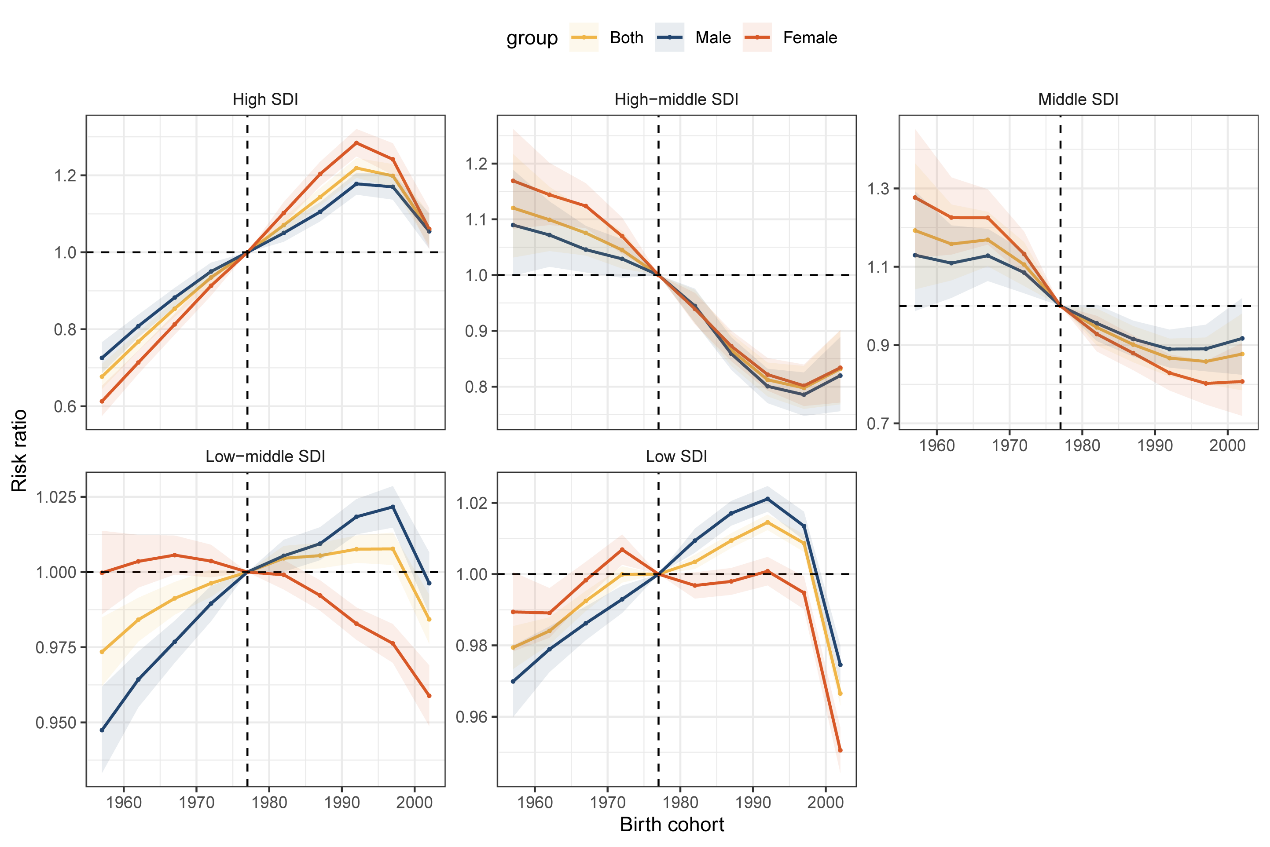
**

**Figure S6** Cohort effects in age-period-cohort models of drug use disorder prevalence in five sociodemographic index quintiles by sex
